# Tocilizumab reverses cerebral vasculopathy in a patient with homozygous *SAMHD1* mutation

**DOI:** 10.1007/s10067-017-3600-2

**Published:** 2017-03-13

**Authors:** Michael Henrickson, Heng Wang

**Affiliations:** 1 Division of Rheumatology, Cincinnati Children’s Hospital Medical Center, 3333 Burnet Ave., Cincinnati, OH 45229-3029 USA; 2 DDC Clinic for Special Needs Children, Middlefield, OH USA

**Keywords:** Aicardi-Goutières syndrome, Cerebral vasculopathy, Moyomoya, *SAMHD1* mutation, Tocilizumab

## Abstract

An auto-inflammatory syndrome consequent to *SAMHD1* mutations involves cerebral vasculopathy characterized by multifocal **s**tenosis and aneurysms within large arteries, moyamoya, chronic ischemia, and early-onset **s**trokes (SAMS). While this condition involves the innate immune system, additional clinical features mimic systemic lupus erythematosus. Mutations in this gene can also cause a subset of the rare genetic condition Aicardi-Goutières syndrome. To date, no established therapy successfully prevents disease progression. We report a corticosteroid-dependent SAMS patient, a 19-year-old male of Old Order Amish ancestry, with diffuse cerebral arteriopathy identified through contrast brain magnetic resonance arteriography (MRA) and MRI. He received subcutaneous adalimumab every 2 weeks for 9 months with minimal response. Then, he started intravenous tocilizumab (6 mg/kg/dose) every 4 weeks. He sustained steadily normalizing cerebral vasculopathy and lab abnormalities resolved, allowing prednisone reduction. We conclude that the cerebral vasculopathy of the homozygous *SAMHD1* mutation-mediated auto-inflammatory disease SAMS responded favorably to tocilizumab infusion therapy.

## Introduction

Cerebral vasculopathy associated with mutations in the *SAMHD1* gene can invoke early-onset stroke [1]. Mutations in this gene can also cause the rare genetic condition, Aicardi-Goutières syndrome (AGS, MIM225750), which bears a phenotypic resemblance to this cerebral vasculopathy occurring in the Amish population. AGS involves increased production of interferon (IFN)-α2 [2]. Loss-of-function mutations in any of six IFN-stimulated genes (*TREX1*, *RNASEH2A*, *RNASEH2B*, *RNASEH2C*, *SAMHD1*, and *ADAR*) are the identified cause of AGS [3]. These mutations involve the innate rather than the adaptive immune system. AGS presents with a phenotypic overlap of early-onset encephalopathy, mimicking congenital viral infection (cerebrospinal fluid lymphocytosis and elevated IFN-α levels, loss of white matter, and basal ganglia calcifications), and features of systemic lupus erythematosus (SLE), including cytopenias, oral ulcers, arthritis, perniosis with cutaneous erythematous lesions, and autoantibodies [4, 5]. AGS can involve a subset of children that develop early-onset SLE [5]. Intracerebral large artery disease and chronic progressive arthropathy with distal joint contractures have been reported in AGS associated with mutation of *AGS5 SAMHD1* protein [6, 7]. Further, AGS associated with heterozygous *TREX1* mutations involves ulcerating acral skin lesions suggestive of chilblain lupus (lupus pernio) [8]. Heterozygous *SAMHD1* mutation can also be associated with progressive arthropathy, distal joint contractures, painful oral ulcers, and chilblains [9].

We describe a patient with a similar clinical spectrum who does not have AGS, rather homozygous *SAMHD1* mutation which led to cerebral vasculopathy. Affected patients within described pedigrees carrying this mutation develop multifocal cerebral stenosis and aneurysms within large arteries, chronic ischemic changes and moyamoya morphology, leading to early-onset strokes [1]. We report the first successful reversal of this cerebral vasculopathy with tocilizumab infusion therapy.

## Case report

The 19-year-old male patient MM, of Old Order Amish ancestry, was previously reported with autosomal recessive homozygous *SAMHD1* mutation (X-28 in the original study) [1]. Briefly, he developed symmetric “dry” polyarthritis at age 9 years, hoarseness at age 12 years requiring vocal nodule resection, bilateral hand and foot pernio at age 13 years, and a clinical course characterized by familial short stature, photosensitivity, and persistent acral vasculopathy presenting as episodic Raynaud’s disease and progressive, bilateral hand and foot sclerodactyly. Peripheral vascular examination revealed diminished bilateral carotid Korotkoff sounds, without bruits of his bilateral carotid or subclavian arteries, or abdominal aorta. Table 1 summarizes salient data from his disease and treatment course.

**Table 1:** Data summary

Elapsed time	6/10/09	8/15/11	1/6/12	5/18/12	7/13/12	8/9/12	11/1/12	1/24/13	5/23/13
Years	0	2.2	0.4	0.3	0.2	0.1	0.2	0.2	0.3
Months	0	26	4.8	4.1	1.9	0.9	2.8	2.8	4
Cumulative (months)	0	26	30.8	34.9	36.8	37.7	40.5	43.3	47.3
Medication (mg/dose)									
Methotrexate (oral, weekly)	20	0	0	0	0	0	0	0	0
Prednisone (oral, daily)	5	10	10	10	60	30	20	10	0
Adalimumab (SC every 2 weeks)	0	40	40	40	0	0	0	0	0
Tocilizumab (monthly infusion number)	0	0	0	0	400 (2nd)	400 (3rd)	400 (6th)	400 (8th)	400 (12th)
Lab results (range)									
Hemoglobin (130–160 g/L)	14.2	159	158	153	140	155	150	144	146
ESR (0–15 mm/h)	27	10	18	47	14	10	10	10	7
Immunoglobulin G (7.24–16.11 g/L)	19.30	18.70	ND	17.20	ND	ND	ND	13.90	ND
Total protein (63–82 g/L)	ND	85	90	91	78	85^a^	84	76	78
Platelets (135–466 × 10^9^/L)	308	248	248	313	232	250	257	253	226
Interleukin-6 (0–0.238 pmol/L)				0.571				1.14	
Joint count									
Active joints	17	0	0	0		0		0	0
Limited joints	29	22	20	20		20		20	17

^*ND* no data^

^a^7/20/12 total protein

**Fig. 1:**
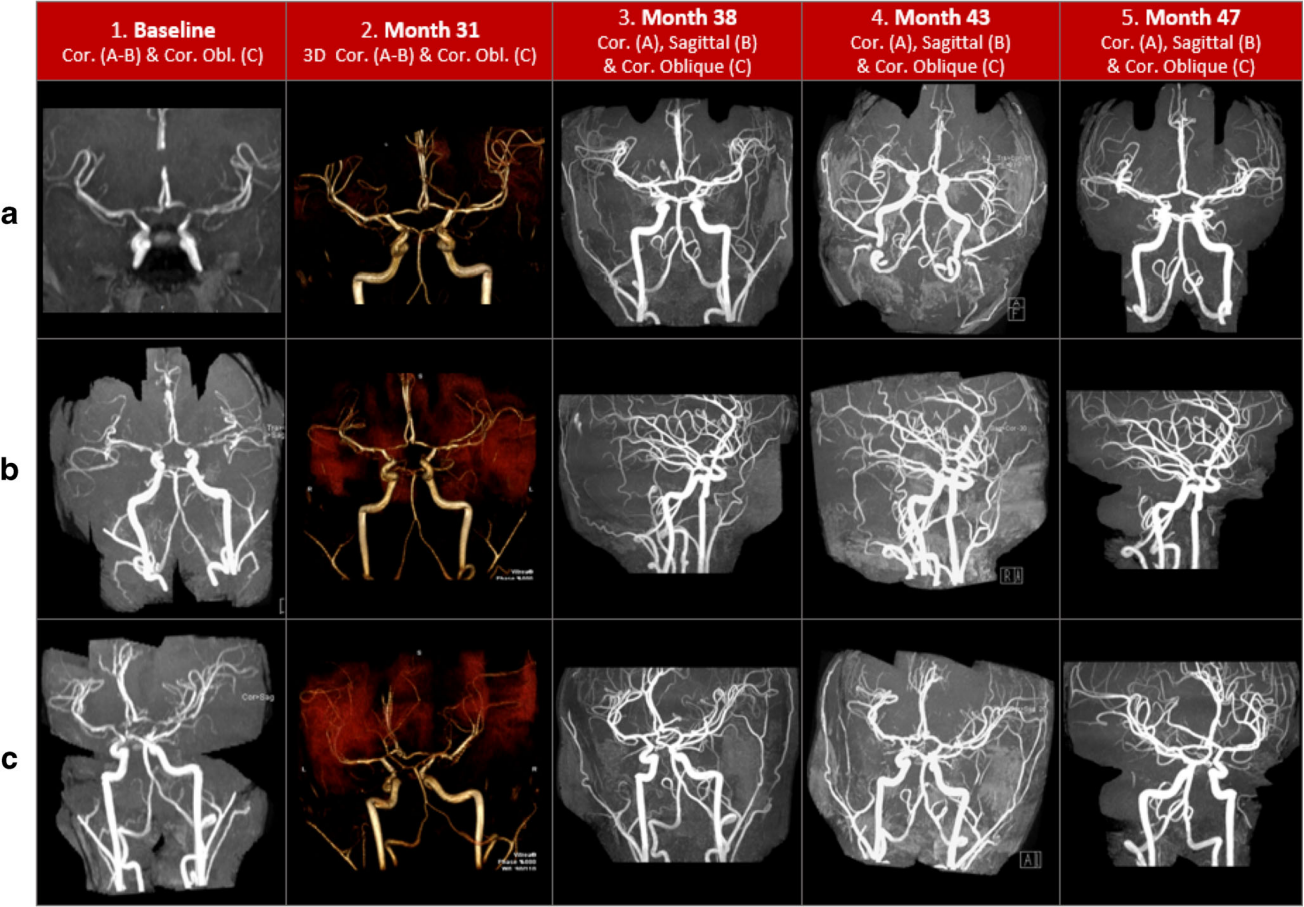
Serial contrast magnetic resonance cerebral arteriography

**Fig. 2:**
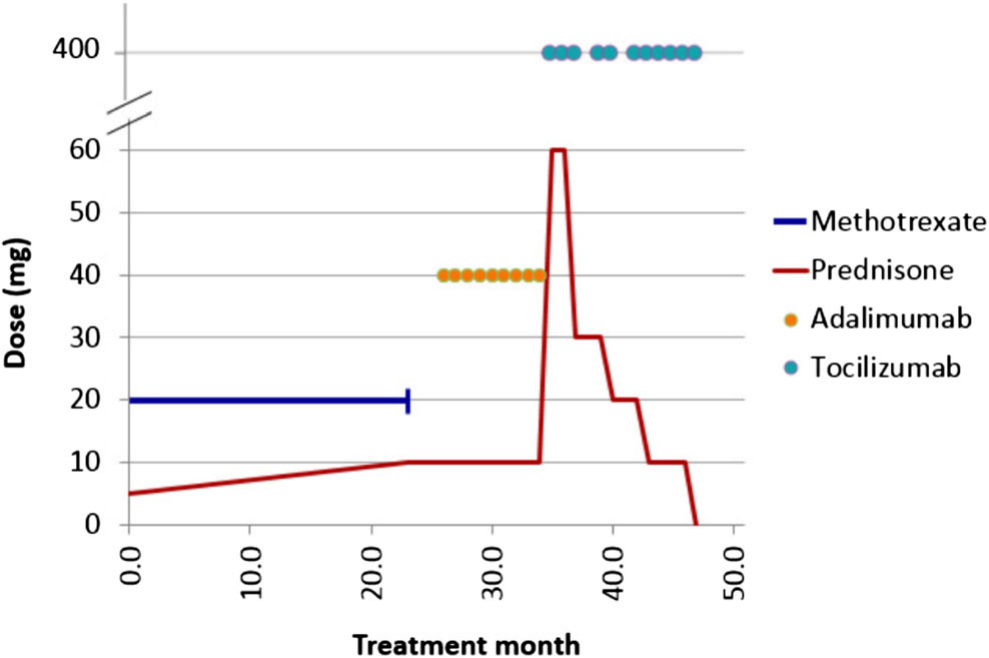
Treatment course

MM underwent contrast brain magnetic resonance arteriography (MRA) and contrast brain MR imaging (MRI) based upon familial risk associated with his sister’s encephalopathy and the desire to identify potential vasculopathy prior to similarly devastating functional consequences. MM’s initial studies performed at age 16 years were abnormal. On MRA, he had diffuse narrowing of his bilateral internal carotid arteries and irregular, narrowed segments of his bilateral anterior cerebral arteries’ A1 and M1 segments (Fig. 1, *1A*–*C*). His bilateral thalamostriate vessels had increased flow, with mild asymmetry in the early arterial phase of the perfusion study, consistent with a compensated perfusion pattern despite extensive fibromuscular changes of arteriopathy. His brain MRI had no evidence of cerebrovascular occlusive disease; however, he had bilateral, diffuse arteriopathic changes of his distal internal carotid, anterior, and middle cerebral arteries. Figure 1 depicts the serial contrast cerebral MRA findings during his treatment course. Figure 2 depicts the temporal sequence of MM’s medication interventions.

Based upon adalimumab’s capacity to penetrate the central nervous system’s blood brain barrier and the putatively protective role of functional *SAMDH1* in mediating the pro-inflammatory tumor necrosis factor (TNF)-α response [1], MM started 40 mg SC adalimumab every other week just prior to obtaining an interval brain MRI/MRA. MM underwent serial, biannual, contrast brain imaging (MRI and MRA) including 3D time-of-flight MRA of the circle of Willis, to assess therapeutic efficacy, and accompanying laboratory biomarkers of inflammation: erythrocyte sedimentation rate (ESR), Immunoglobulin G (IgG) and total protein. Throughout his pre-biologic or biologic therapy clinical course, the C-reactive protein never increased, nor did he manifest anemia of chronic disease.

Compared to his baseline study (month 0), contrast brain MRI and MRA obtained at onset of adalimumab therapy (month 26) indicated a mixed picture. Vasculopathy progressed with mild narrowing of his vertebral arteries’ distal segments and bilateral globus pallidus early collateralization consistent with moyamoya. Cerebrovascular disease remained unchanged with persistent bilateral distal internal carotid artery narrowing and indicated mild improvement of the anterior cerebral A1 segments and proximal middle cerebral arteries (M1 segments). There were equivocal changes of mild basilar artery narrowing and subtle abnormalities of the internal capsules’ posterior limb (arising from the anterior cerebral A1 and M1 segments). There was also increased signal intensity of the corticospinal tracts, extending into his cerebral peduncles and pons. The second study obtained during adalimumab therapy at month 31 indicated stable vasculopathy without progressive change in these previously identified abnormalities (Fig. 1, *2A*–*C*).

During a 9-month course of adalimumab therapy, there was mild evidence of disease advancement (including evolving moyomoya) but predominant stability while he remained on a daily 10-mg prednisone dose. However, the therapeutic goal was amelioration of identified extra- and intracranial medium- and small-vessel vasculitis with elimination of corticosteroid dependence. Failure to meet these goals compelled the decision to change biologic therapy. Serum interleukin-6 (IL-6) was elevated (0.571 pmol/L) [normal 0–0.238] after 9 months of adalimumab therapy. With this evidence, he discontinued adalimumab at month 35 and started tocilizumab 400 mg IV every 4 weeks at month 36 (6.2 mg/kg/dose).

Although MM did not appreciate a clinical change in his perniosis or articular disease after his first two monthly tocilizumab infusions, he had appreciable improvement in his cerebral vasculopathy. This coincided with temporal deterioration in unilateral hand perniosis requiring escalation in his prednisone dose to 1 mg/kg/day during his second month of tocilizumab therapy. He tapered prednisone successfully to 0.5 mg/kg/day by his third monthly infusion, when he obtained repeat brain imaging. Following two tocilizumab infusions, the MRA at month 38 indicated similarly stable findings with the exception of normalization of his A1 and M1 cerebral artery segments (Fig. 1, *3A*–*C*). At month 43, the second MRA obtained during tocilizumab therapy indicated stable increased signal intensity within the bilateral internal capsules’ posterior limbs; other previous abnormalities remained stable, and the A1 and M1 cerebral artery segments stayed normal (Fig. 1, *4A*–*C*). At month 47, repeat MRA following 11 consecutive tocilizumab infusions revealed stable, bilateral distal internal carotid artery narrowing, continued A1 and M1 segment improvement, resolution of globus pallidus communicating artery collateralization (moyamoya), and normalization of the previously increased bilateral cortical spinal tract signal intensity (Fig. 1, *5A*–*C*).

## Discussion

The auto-inflammatory disease SAMS, an acronym for cerebral stenosis, aneurysm, moyomoya, and stroke, produces a heterogeneous phenotype of diverse clinical presentations, including cerebral palsy, stroke, developmental delay, failure to thrive, chilblains, and arthritis. Early features include mild intrauterine growth restriction, infantile hypotonia, and irritability, followed by failure to thrive and short stature. During cold weather months, affected patients experience a spectrum of vascular abnormalities of their acral regions including Raynaud’s disease and chilblain lesions (lupus pernio). They may also have a low-pitch hoarse voice, glaucoma, migraine headache, and arthritis characterized by joint tenderness and restricted motion. Patients do not have hypertension; their cardiopulmonary, hepatic, and renal function is normal. The elevated ESR, IgG, neopterin, and tumor necrosis factor-α (TNF-α) found in these patients are consistent with inflammatory disease. Cerebral vasculopathy is a major hallmark found in all affected individuals typified by stenoses and aneurysms. Distinctive neuroimaging findings include chronic ischemic changes, multifocal stenoses of the large intracranial arteries, including some with moyamoya morphology and evidence of prior acute infarction and hemorrhage [1]. Clinical outcomes in affected patients are largely dependent on cerebral vasculopathic progression. Indeed, the early-onset or recurrence of cerebral vasculopathy-associated strokes always predicts severe functional impairment and poor cognitive outcomes.

While the specific pathophysiology is uncertain, aberrant processes involving the innate immune system may support one or several potential mechanisms. Defective metabolism of intracellular nucleic acids can activate a cell-intrinsic autoimmune response, pivotal to host capacity to discriminate between self and non-self (e.g., viral) nucleic acids. Nucleic acid recognition is an essential innate immune strategy for detecting viral infection. Functional regulation involves prevention of self-activated innate immunity, including negative regulation of the IFN-stimulatory DNA (ISD) response. Virtually all IFN-mediated antiviral immunity occurs via two types of nucleic acid detection systems: toll-like receptors (TLRs) and cytosolic sensors [10]. Because flawed distinction of viral from self nucleic acids can occur, defective clearance of self-derived nucleic acids may lead to severe, IFN-associated autoimmunity [11]. Activation of TLRs on autoreactive B cell lymphocytes is a major contributing mechanism. Specific TLRs predispose to autoantibody production in murine models of lupus and autoimmunity. Such activation of TLR-dependent IFN production also occurs in human autoimmunity (e.g., psoriasis) [12]. In each circumstance, accumulated nucleic acids are detected by non-cell autonomous mechanisms rather than cell-dependent detection [11].

Cytosolic nucleic acid sensors can initiate autoimmunity if flawed control mechanisms exist. Negative regulators of the ISD response play a major role in preventing self-activation of innate immunity via cell-intrinsic components. One important negative regulatory mechanism in part involves 3′ repair exonuclease 1 (*TREX1*) induction. *TREX1* is a cytosolic toll-like receptor-independent, antiviral pathway that detects DNA and triggers immune activation through transcription factor IFN regulatory factor 3 [11]. Cell-intrinsic initiation of autoimmunity has distinct requirements for regulation and unique mechanisms that precipitate lymphocyte-dependent autoimmunity. Autoimmunity may be triggered by cell-intrinsic initiation of the ISD pathway in *TREX1*-deficient mice, preceding a TLR-dependent contribution to autoantibody production [11].

Disease pathogenesis may reflect *SAMHD1* protein loss of function instead of partially functional protein expression [1]. The *SAMHD1* gene mutation localizes to chromosome 20q11.22-q12 involving a pathogenic, homozygous splice-acceptor site mutation (c.1411–2A > G) in intron 12. The *SAMHD1* gene consists of 16 coding exons and encodes a protein of 626 amino acids. This identified mutation results in exon 13 mRNA transcription skipping, leading to formation of an aberrant protein. There are two functional domains in the *SAMHD1* protein: a sterile alpha motif (SAM) (residuals 42–110) and an HD domain (residuals 160–325) [1]. The SAM domain has the ability to bind RNA [13]. The HD domain recurs in an enzyme superfamily with phosphohydrolase activity which is involved in nucleic acid metabolism [14]. *SAMHD1* is a dGTP-stimulated triphosphohydrolase; it converts deoxynucleoside triphosphates (dNTP) to deoxynucleoside and inorganic triphosphate. This gene decreases dNTP to minimal levels that do not support reverse transcription, preventing viral infection [15, 16]. *SAMHD1* also plays a significant role in clearance of cellular waste and maintaining IFN homeostasis [2]. While this suggests *SAMHD1* may function as a nuclease, *SAMHD1*, like *TREX1*, is also another potential negative regulator of the ISD response. Both genes may have a similarly protective, immunomodulatory role in preventing self-activation of innate immunity. Finally, *SAMHD1* may further provide a protective role in mediating TNF-α pro-inflammatory responses [2].

In a case-control study of AGS, a phenotypically similar inflammatory disorder caused by mutations in any of six IFN-stimulating genes (*SAMHD1, TREX1*, *RNASEH2A*, *RNASEH2B*, *RNASEH2C* and *ADAR*), 90% (74/82) of affected patients vs. 7% (2/29) of controls had a positive IFN score, reflecting increased expression of these genes [3]. Clinically, AGS presents as an infantile encephalopathy, manifesting as progressive microcephaly and psychomotor retardation; 25% of patients die in early childhood consequent to its leukodystrophy and microangiopathy. A leading hypothesis regarding AGS is that the accumulation of intracellular nucleic acids triggers an auto-inflammatory response and consequent increased astrocytic IFN-α production [17]. In AGS, chronic IFN-α exposure results in altered gene expression for proteins involved in the stability of brain white matter (ATF4, eIF2Bα, cathepsin D, cystatin F), an increase of antigen-presenting genes (human leukocyte antigen class I) and downregulation of pro-angiogenic factors and other cytokines, i.e., vascular endothelial growth factor and interleukin-1 (IL-1). These effects on therapeutic targets for AGS and other IFN-α-mediated encephalopathies, may primarily involve downstream IFN-α signaling cascade effectors rather than only IFN-α [17].

The relationship of increased IL-6 production and IFN-α signaling in the context of *SAMHD1* mutation disease is unclear. However, the model of viral infection provides context for indirect evidence of increased IL-6 expression consequent to IFN-α priming. During viral infection, host cells are alerted by the presence of circulating class I IFN, preparing them for response if they become infected. When a cell recognizes double-stranded RNA (dsRNA), this indicates the cell is likely already infected with virus. However, when a cell encounters either IL-1β or TNF-α, the cell receives warning about the host’s escalating inflammatory response. This warning does not indicate direct viral infection of the cell. Instead, the class I IFN priming phenomenon promotes a vigorous response to direct viral infection more than IL-1β or TNF-α provide. While IFN-α does not directly induce IL-6 expression, prior cellular exposure to IFN-α synergistically enhances IL-6 expression in response to the viral dsRNA. The magnitude and kinetics of IL-6 induction vary in these situations since class I IFNs selectively enhance response to dsRNA [18].

Systemic vasculitides with increased IL-6 expression include Takayasu’s arteritis [19–22], acute Henoch-Schönlein purpura [23], giant cell arteritis [24, 25], acute Kawasaki disease [26–28], and various large vessel vasculitides [29–33]. Tocilizumab therapy provides a promising role in achieving disease remission for refractory neuro-Behçet’s disease, relapsing or refractory giant cell arteritis, and large vessel vasculitis secondary to Takayasu’s arteritis, Cogan syndrome, and relapsing polychondritis [29, 33–39].

TNF inhibition (typically infliximab) has circumstantial evidence suggesting efficacy in open label use for large vessel vasculitis secondary to refractory Takayasu’s arteritis, relapsing polychondritis, Behçet’s disease (also including adalimumab), and relapsing polychondritis. However, this approach has not been effective for large vessel vasculitis due to giant cell arteritis as demonstrated in randomized controlled trials or a prospective trial for granulomatosis with polyangiitis [31, 40]. Adalimumab stabilized our patient’s cerebral vasculitis without remarkably reversing progressive arteriopathy.

Results are mixed regarding the efficacy of TNF inhibitors for the therapy of lupus pernio. TNF inhibitors can induce this disease [41]. However, lupus pernio secondary to sarcoidosis responded swiftly to adalimumab in one case report [42]. Tocilizumab has not been reported for this use. Neither adalimumab nor tocilizumab were effective for our patient’s SAMS-associated pernio.

## Conclusions

There is no established, uniformly successful treatment strategy for SAMS, an auto-inflammatory disease consequent to homozygous *SAMHD1* mutation. Relapses remain common with prednisone tapering. Traditional corticosteroid-sparing, non-biologic immunosuppressive medications used in the treatment of rheumatic diseases involving adaptive immunity, e.g., methotrexate, are ineffective in the treatment of SAMS cerebral vasculopathy, a disease involving the innate immune system. Based on the effectiveness of tocilizumab in controlling a variety of active large vessel vasculitides including Takayasu’s arteritis and giant cell arteritis, tocilizumab provides a novel therapeutic option for SAMS. Following its initiation, MM obtained disease control within 2 months, followed by substantial reversal of much of his cerebral vasculopathy after nearly a year of tocilizumab treatment. With this apparent efficacy and durability, tocilizumab may offer a means of controlling cerebral vasculopathy from the auto-inflammatory disease SAMS.
